# Practical Thoughts

**Published:** 1863-05

**Authors:** 


### Editorial.

### "PRACTICAL THOUGHTS."

The article in the present number of the Register, with the above caption, is quite suggestive, and we can not refrain from a few remarks upon it. Though it is good, there are perhaps a few things in it with which all will not agree.

We are really sorry for the demise of the Mad River Dental Society, and we very much regret to aid in giving publicity to the fact, but it is to be hoped it will not produce discouragement elsewhere. Expectation has looked to this Society as one from which much good would emanate to the profession, and especially to those under its immediate influence. We do not know of what it died, but it seems to have foundered on a *fee bill*, at least we would infer that from the "*Practical Thoughts.*"

Dr. P. thinks it impossible to establish a uniform rate of fees among dentists, because there is so great a diversity of professional *character* and *ability*. Now, while it is true that this diversity exists, and that those who possess and exercise the largest ability will always command the higher appreciation and fee; yet it is competent and practical to establish a minimum rate of fees, up to which *all* may come. Operations performed upon the teeth in such a manner as to secure the object sought, if they are good honest operations, should command the same fee for one as for another. Some, of course, possess more skill, and when circumstances will justify, will exercise it; hence arises the larger fee.

In an Association of Dentists the presumption is that all can do *efficient* work, and that thus far all are alike entitled to reward, and it is upon this basis that fee bills are formed. In the establishment of fee bills there is mutual advantage to all concerned—both to the *people* and to the *profession.* To the former because they are thereby more certain to obtain good and efficient operations; and the latter because they are thereby stimulated to a higher order of skill and execution, and those who have been supposed, or thought themselves to have been somewhat behind, are thus brought forward and placed abreast with the best. He then has an opportunity to go on and make higher attainments in his chosen science and art without being cramped and throttled by beggarly fees. Men in the dental profession will stultify themselves in such a way as those in no other occupation or calling in life will do. Employ a hod carrier at two dollars per day and it would be rather a difficult matter to employ another at one dollar per day to work with him—and why? He would reply, My services are worth as much as that of any other man. His self-respect is touched, and he is insulted. Yet men in the dental profession, claiming for it honor and dignity, will do that which would insult hod carriers and street scavingers—hold out low charges as an inducement for patronage.

He who has been exercising his calling for far less remuneration than he ought, should be thankful to any who would assist him out of the difficulty, and he should manifest his appreciation of the kindness by steadfastness and progression, and not by stealthily *sliming* back into the slough from which he has been extricated. The adoption of a uniform rate of fees is an instrumentality upon which, to a considerable extent, progress and improvement in the profession depends, for those who are all the time running a tilt upon each other in regard to charges will not associate and aid each other professionally, and that, as all know who have any experience in the matter, is one of the greatest *sharpeners.* The public will always suspect those who are suspecting, berating and underrating one another.

The comparison which Dr. P. makes in regard to the fees for artificial teeth, and operations upon the natural ones, we can scarcely regard as just. While we agree with him that almost universally the charges for operations upon, and treatment of, the natural teeth are far less than they should be, we would by no means conceed that the charges for artificial teeth are too high, but on the contrary, that they too are less than they should be. Dr. P. speaks of substitutes for the natural teeth as a mere mechanical matter throughout. Now, this is certainly somewhat aside from the mark; for in the first place, in the construction of artificial dentures, the science and skill of the maker is continually drawn upon because of the great variety and diversity of the dentures, for scarcely ever does the dentist make two pieces in all respects alike; and hence it can not be, if properly done, a mere mechanical operation.

And then again, in the adaptation and introduction of artificial dentures, physiological and pathological conditions and predispositions must be regarded and considered, and to do this properly they must be understood. The mere mechanic knows nothing of these.

Again, to select and arrange teeth is a work requiring the highest artistic skill, such as no mere mechanic possesses. It is true that the laboratory workman can do the principal part of the hand work in getting up a set of teeth, such as moulding, melting, casting, swaging, grinding, soldering, &c., but the accomplishment of all this does not make a set of teeth. The selection of the appropriate material for a base, the procuring a perfect impression, the proper adaptation of a denture in the mouth, in view of the physiological and pathological indications, requires as much nice discrimination, and as deep and enlarged knowledge, as for the performance of operations upon the natural teeth.

We opine if Dr. P.'s plan was carried out it would work fearfully against his favorite department, for reduce the expense of artificial teeth down to a mere pittance and the greater majority of people will give no attention to the preservation of their natural teeth, and especially after they have become diseased and painful. They will reason thus: Let my teeth go as soon as possible, the sooner the better; and then for a small sum I will get a nice set of artificial teeth that will be as good as natural, and never pain me. Now, this is the reasoning since the cost of rubber-work is reduced to a mere pittience ; and it would be still more general if all kinds of artiffcial dentures were reduced in price as Dr. P. suggests. The adults would neglect their teeth, children's teeth would be neglected, and the tendency would be to general ruin.

It rather seems that every proper consideration would suggest that an advance should be made in artificial work. A good set of teeth is always worth more to a patient than it costs, and an inferior set is dear at any price. The natural teeth will be appreciated and valued, as artificial substitutes are more expensive and difficult to obtain. The people will then demand that the natural teeth be preserved, and dentists must then come up to the point of attainment to enable them to accomplish that object. The true dentist will make it his chief aim to preserve the natural teeth rather than to sacrifice them with the view of making artificial substitutes. Yet there are dentists all over the country pursuing an opposite course—encouraging the loss of the natural teeth that they may supply their place with the artificial.

But then comes the question, What is to be done to remedy the difficulty? This question we propose to answer in the next number. T.

